# Defining the Qualities of High-Quality Palladium on Carbon Catalysts for Hydrogenolysis

**DOI:** 10.1021/acs.oprd.0c00536

**Published:** 2021-06-23

**Authors:** Conor J. Crawford, Yan Qiao, Yequn Liu, Dongmei Huang, Wenjun Yan, Peter H. Seeberger, Stefan Oscarson, Shuai Chen

**Affiliations:** † Centre for Synthesis and Chemical Biology, University College Dublin, Belfield, Dublin, Ireland; ‡ Department of Biomolecular Systems, Max Planck Institute of Colloids and Interfaces, 14476 Potsdam, Germany; § Center of Materials Science and Optoelectronics Engineering, University of Chinese Academy of Sciences, Beijing 100049, People’s Republic of China; ∥ State Key Laboratory of Coal Conversion, Institute of Coal Chemistry, Chinese Academy of Sciences, Taiyuan 030001, People’s Republic of China

**Keywords:** heterogeneous catalysis, global deprotection, glycans, total synthesis

## Abstract

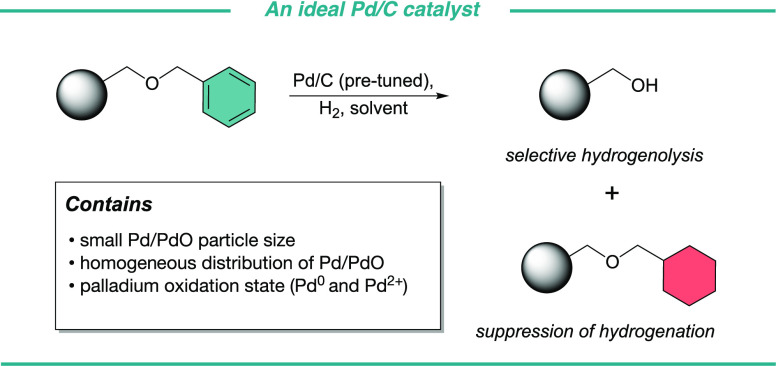

Palladium-catalyzed hydrogenolysis is often the final step in challenging natural product total syntheses and a key step in industrial processes producing fine chemicals. Here, we demonstrate that there is wide variability in the efficiency of commercial sources of palladium on carbon (Pd/C) resulting in significant differences in selectivity, reaction times, and yields. We identified the physicochemical properties of efficient catalysts for hydrogenolysis: (1) small Pd/PdO particle size (2) homogeneous distribution of Pd/PdO on the carbon support, and (3) palladium oxidation state are good predictors of catalytic efficiency. Now chemists can identify and predict a catalyst’s efficiency prior to the use of valuable synthetic material and time.

## Introduction

Palladium-catalyzed hydrogenolysis is often the ultimate step in challenging total syntheses to remove ether protecting groups (e.g., benzyl or naphthylmethyl ethers) to yield the desired target compound. While deceptively simple, this final step is often a major bottleneck. This challenge is often encountered when deprotecting synthetic oligosaccharides, as many benzyl ethers (>30 groups) must be removed simultaneously, in high yields, high selectivity, and short reaction times. The transformation of a highly lipophilic molecule into a hydrophilic one also poses a range of solubility issues.

Many practitioners of carbohydrate chemistry have experienced long reaction times, poor yields, and saturation of aromatic protecting groups. Recent examples of global deprotection of large polysaccharides well illustrate this challenge. For example, the Yu group completed a total synthesis of a 128-mer^1^ that ended with a 15% yield in the hydrogenolysis reaction. Reports of the automated glycan assembly of Lewis type antigens,^2^ and the largest glycan synthesized to date, a 151-mer,^3^ reported final deprotection yields ranging from 17–54% depending on the glycan. In the synthesis of glycans related to *Cryptococcus neoformans*, glucuronoxylomannan (GXM), naphthoxylosides, and high mannose N-glycans, saturation of aromatic protecting groups to saturated ethers has been reported.^4−8^ Separation of these saturated side products from the desired compound complicates the final purification step. To overcome these selectivity issues, we introduced a catalyst pretuning methodology (dimethylformamide (DMF):H_2_O, 37% HCl) that increases catalyst selectivity toward hydrogenolysis rather than hydrogenation through amine poisoning. The catalyst pretreatment inhibits these unwanted saturation by-products and gives access to pure synthetic oligosaccharides.^4,9,10^ This methodology successfully tackled an issue we faced (catalyst selectivity) but another key question was how and why different palladium on carbon (Pd/C) catalysts lead to such variable results. Pd/C catalysts remain a “black box” and force extensive testing on complex materials to identify efficient catalysts, defined under the parameters of short reaction times, high isolated yields, and its selectivity toward hydrogenolysis over hydrogenation.

To avoid such extensive testing, we sought to better understand the key differences between commercial sources of Pd/C, to identify high-quality catalysts rapidly in the future. Ultimately, we envisage this could allow for prediction of palladium on carbons quality—prior to use of valuable time and synthetic material. Furthering our understanding of what makes a Pd/C catalyst optimal will allow the design of more attractive heterogeneous catalysts.

Here we demonstrate clues to a palladium catalysts’ efficiency can be found by studying its surface chemistry. A combination of high-resolution transmission electron microscopy (HRTEM), X-ray photoelectron spectroscopy (XPS), N_2_ adsorption and desorption (Brunauer-Emmett-Teller, BET), and X-ray diffraction (XRD) analysis was used to define the properties of high-performance catalysts. Giving chemists a framework to compare and assess the quality of a catalyst at hand with the goal of circumventing the need for extensive optimization experiments with valuable material from synthesis. The key parameters for an effective catalyst include: small Pd/PdO particle size, homogeneous distribution of Pd/PdO on the carbon support, and palladium oxidation state.

## Results and Discussion

**Scheme 1:**
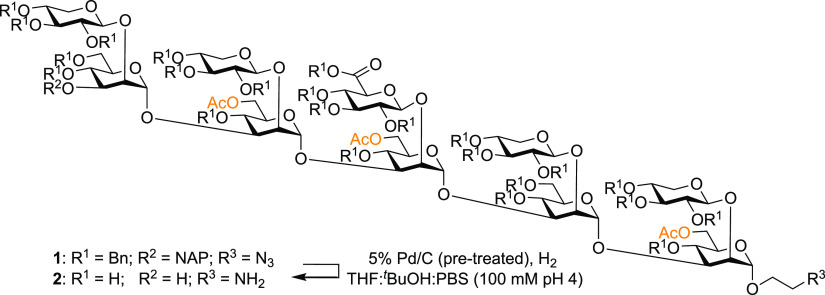
Global Deprotection of Serotype A Decasaccharide

Our optimization study used a synthetic decasaccharide **2** (Scheme 1).^11^ Representing a challenging substrate as it assumes a branched tertiary structure^9^ and contains 25 groups that need to be reduced under a hydrogen atmosphere, including benzyl ethers, naphthylmethyl ethers, and azides.

### Catalytic Performance of Commercial Catalysts

Pearlman’s catalyst (20% Pd[OH]_2_/C, Sigma-Aldrich) and 10% Pd/C (Sigma-Aldrich) led to exceedingly long reaction times (5–6 days) (Table 1, Entries 1, 2, 7, 8), provided intermediate yields (57–66%), and high levels of saturation of aromatic protecting groups (39–53%) that could not be separated from the desired product. While using the 5% Pd/C catalyst (Strem Chemicals, Table 1, Entries 3 and 9) we found that reaction times were the shortest (1.5–2 days), yields were the highest (82–84%), and we detected the lowest levels of saturation of aromatic protecting groups (10%). We did not observe major effects of the solvent in hydrogenolysis on the isolated yields but did observe minor changes in reaction times and catalyst selectivity (Table 1, Entries 1–3, 7–9).

**Table 1:** Global Deprotection of GXM Glycan

entry	substrate	catalyst	supplier	pretreatment	level of saturation of side products [%]	time [days]	yield [%]
1	**1**	20%Pd[OH]_2_/C	Sigma-Aldrich	no^a^	39	5.5	66^b^*
2	**1**	10% Pd/C	Sigma-Aldrich	no^a^	53	4	57^b^*
3	**1**	5% Pd/C	Strem Chemicals	no^a^	10	1.5	84^b^*
4	**1**	20%Pd[OH]_2_/C	Sigma-Aldrich	yes^c^	0	6	66
5	**1**	10% Pd/C	Sigma-Aldrich	yes^c^	0	5	58
6	**1**	5% Pd/C	Strem Chemicals	yes^c^	0	2	88
7	**1**	20%Pd[OH]_2_/C	Sigma-Aldrich	no^d^	36	6	67^b^*
8	**1**	10% Pd/C	Sigma-Aldrich	no^d^	54	4	57^b^*
9	**1**	5% Pd/C	Strem Chemicals	no^d^	9	2	82^b^*

a Untreated catalyst, EtOAc/MeOH/AcOH (4:1:1:1 v/v/v), 10 bar, and rt.

b Combined yield of desired decasaccharide and saturated side products.

c Preconditioned catalyst (see protocol), THF/^*t*^BuOH/PBS (phosphate-buffered saline) (100 mM pH 4) (60:10:30 v/v/v), 10 bar, and rt.

d Untreated catalyst, THF/^*t*^BuOH/PBS (phosphate-buffered saline) (100 mM pH 4) (60:10:30 v/v/v), 10 bar, and rt. * this yield includes inseparable amounts of cyclohexyl methyl ether side products.

**Figure 1:**
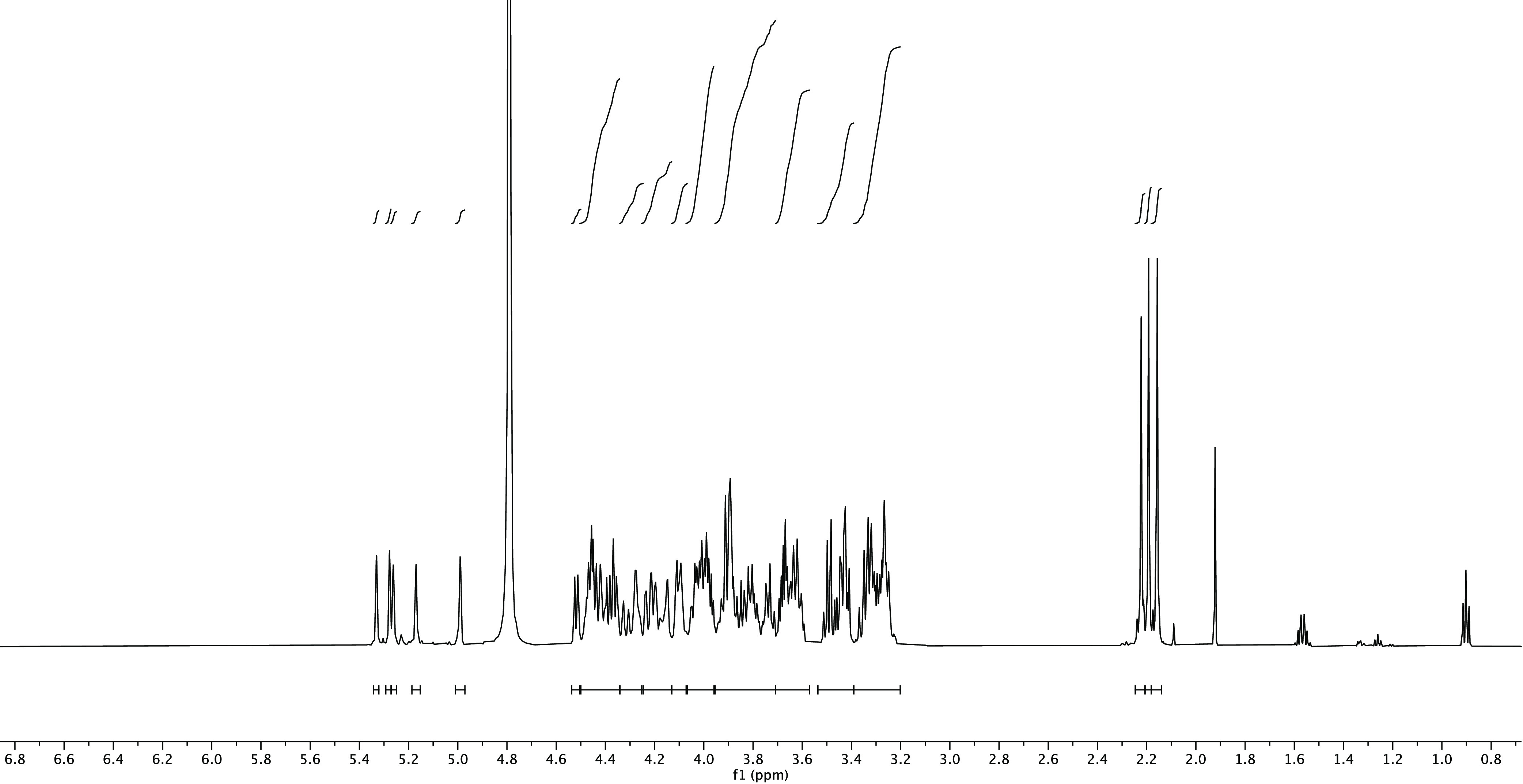
^1^H NMR spectrum of serotype A decasaccharide.

Having shown that both pH and hydrogen pressure have negligible effects on catalyst selectivity,^4^ we experimented hydrogenolysis reactions using our recently disclosed catalyst’s pretuning strategy.^4^ This protocol is useful as it inhibits saturation of aromatic protecting groups (such as benzyl and naphthylmethyl ethers). Using this approach, no saturation of aromatic protecting groups occurred and the desired 6-*O*-acetylation pattern stayed intact (Table 1, Entries 3–6).^4^ Reaction times using the pretreated catalyst were similar to those of the nontreated catalysts (Table 1). Overall, we found that 5% Pd/C (Strem Chemicals) allowed for access to the desired decasaccharide **2** in the shortest reaction times (2 days), highest yields (88%), and no aromatic protecting group-related saturation but only when using the pretuning methodology (Figure 1, Table 1, Entry 3).^4^

To improve our understanding of the wide variability experienced when using different palladium on carbon catalysts, we characterized the catalysts with a range of spectroscopic and imaging techniques. Given that each palladium catalyst did not differ significantly in activity (reaction times and yields) as a result of the preconditioning process, we chose to first analyze the catalysts prior to pretreatment.

### Characterization of Palladium on Carbon Catalysts

#### X-ray Diffraction (XRD)

**Figure 2:**
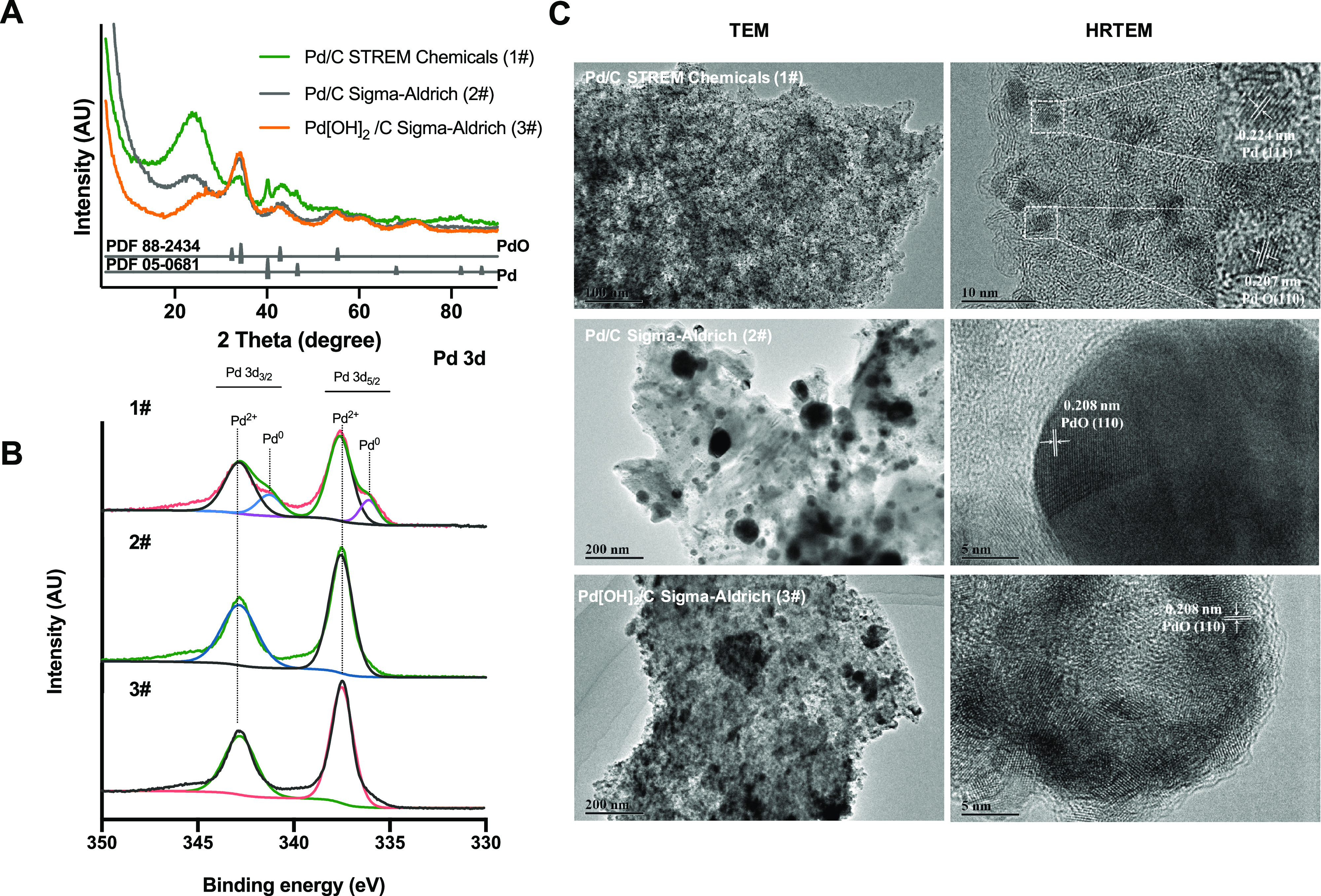
Characterization of palladium on carbon catalysts. Pd/C from STREM Chemicals (1#), Pd/C from Sigma-Aldrich (2#), and Pd[OH]_2_/C from Sigma-Aldrich (3#). (A) XRD patterns of Pd/C. (B) XPS for Pd 3d electrons. (C) TEM and HRTEM of the catalyst. Scale inset.

XRD analysis of the most efficient catalyst (1#, 5% Pd/C from Strem Chemicals) (Figure 2A) obtained diffraction peaks at 2θ of 33.3, 34.4, 42.9, and 55.3° that are assigned to the (002), (101), (110), and (112) facets of tetragonal PdO (powder diffraction file, PDF No. 88-2434), respectively.^12,13^ The existence of palladium was confirmed by the peaks at 2θ of 40.1, 46.7, and 68.1° that correspond to the (111), (200), and (220) planes of cubic Pd (PDF No. 05-0681), respectively.^14−16^ While the XRD pattern for the two lower quality catalysts (Figure 2A, 2# and 3#) show clear peaks at 33.3, 34.4, 42.9, and 55.3° for crystalline tetragonal PdO, matching well with PDF No. 88-2434 but no Pd was detected. The intensities of the corresponding XRD peaks from the two lower quality catalysts (2# and 3#) showed a significant increase, compared to that of the best catalyst (1#), confirming the higher PdO content in the lower quality catalysts. However, the wide half-peak width of PdO signified the poor crystallinity/small crystal particle sizes in all commercial samples. Additionally, all samples showed a broad peak located at 2θ of ∼25°, which was assigned to the (002) diffraction planes of graphite microcrystals in the disordered carbon.^16,17^

The presence of large quantities of PdO (Pd^2+^) in the two lower quality Pd/C catalysts (Sigma-Aldrich catalysts, Table 1, Entries 1–2 and 4–5) in combination with the larger particle sizes likely contributes to the longer reaction times required when using these catalyst batches. It is also important to consider the steric bulk of the glycan under deprotection, meaning that smaller palladium particle sizes can more favorably interact and perform catalysis—leading to the faster observed deprotection times.

When Pd/C catalysts containing only PdO (both Sigma-Aldrich catalysts) are first exposed to the hydrogen atmosphere, they must first be reduced from Pd^2+^ to Pd°, meaning that the oxidative addition step in the catalytic cycle cannot initially occur (meaning lower quantities of active Pd are present to complete hydrogenolysis). Larger Pd particles are well understood to affect rates of the reaction and explain the lower efficiency of the Sigma-Aldrich catalysts.^18^ Our data indicates that higher percentage loading of Pd does not necessarily correspond to higher reaction rates.

### Transmission Electron Microscopy (TEM)

TEM images were taken of each catalyst to visualize the morphology and size distribution of the catalysts (Figure 2C). The most effective catalyst (5% Pd/C Strem Chemicals) indicated that Pd and PdO nanoparticles are uniformly dispersed on the carbon with the mean size of ∼4 nm. The existence of many active sites in the corners and edges of small-sized nanoparticles is consistent with the observation of a more favorable catalytic performance during the hydrogenolysis reactions (Table 1 Entries 3 and 6).

The high-resolution TEM (HRTEM) image of the 5% Pd/C (Strem Chemicals) (Figure 2C) reveals two lattice fringes with the spaces of 0.224 and 0.207 nm, which correspond to the (111) crystalline plane of Pd and (110) crystalline plane of PdO, respectively. This indicated the coexistence of Pd and PdO in the high-quality catalyst (1#). Conversely, the low-quality catalysts (2# and 3#) exhibited large particle sizes, as a result of severe particle agglomeration and poor size distribution, which is not favorable for the catalytic process, corresponding to the lower isolated yields and longer reaction times (Table 1 Entries 1 and 2). The low-quality HRTEM image (Sigma-Aldrich) indicated that the lattice spacing of ∼0.208 nm corresponds to the (110) crystal plane of PdO.

### X-ray Photoelectron Spectroscopy (XPS) Analysis

The elemental constituents and states of the catalysts were analyzed using high-resolution XPS (Figure 2B). Binding energies of 337.5 and 342.8 eV were observed in all catalysts and were ascribed to Pd^2+^ 3d_5/2_ and 3d_3/2_ split orbitals of PdO, respectively. Additionally, catalyst 1# contained two lower binding energies of 336.0 and 341.1 eV, which were assigned to 3d_5/2_ and 3d_3/2_ levels of metallic Pd (Pd^0^), respectively.^19−21^ These findings confirm that both PdO and Pd exist in the most active catalyst (5% Pd/C, Strem Chemicals) but not in the other two catalysts.

### N_2_ adsorption and desorption (Brunauer-Emmett-Teller, BET)

**Figure 3:**
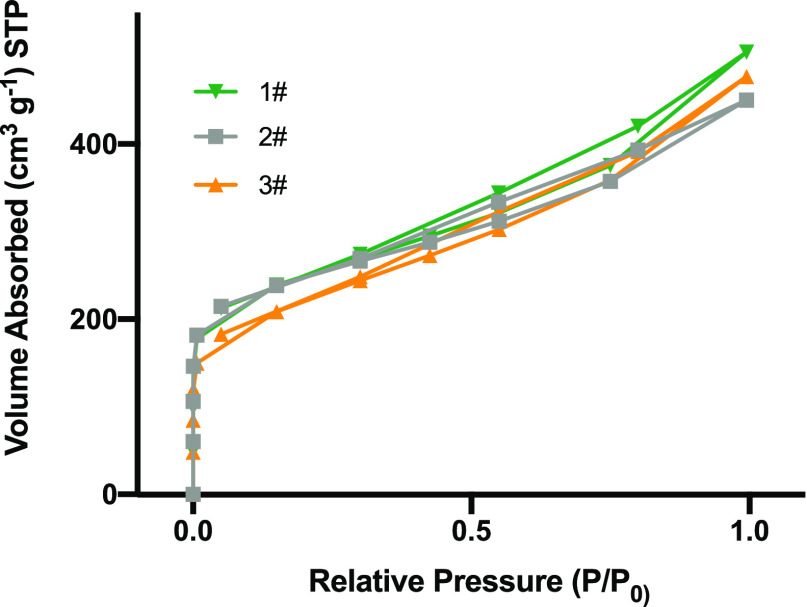
N_2_ adsorption–desorption isotherms of samples Pd/C STREM Chemicals (1#), Pd/C Sigma-Aldrich (2#), and Pd[OH]_2_/C Sigma-Aldrich (3#).

To verify the effect of catalyst microstructure on catalytic performance, the specific surface area and microstructure of the catalysts were investigated by N_2_ adsorption/desorption isotherms (Figure 3). The isotherms display a typical type IV behavior, with a sharp uptake at low relative pressure, which is distinctive of mesoporous materials, suggesting that plenty of mesopores exist in these catalysts; this conclusion is also supported by TEM characterization. The Brunauer–Emmett–Teller (BET) specific surface areas of the samples 5% Pd/C from Strem Chemicals (1#), 10% Pd/C from Sigma-Aldrich (2#), and 20%Pd[OH]_2_/C (3#) are about 897, 898, and 778 m^2^/g, respectively, with none of the catalysts differing significantly.

**Figure 4:**
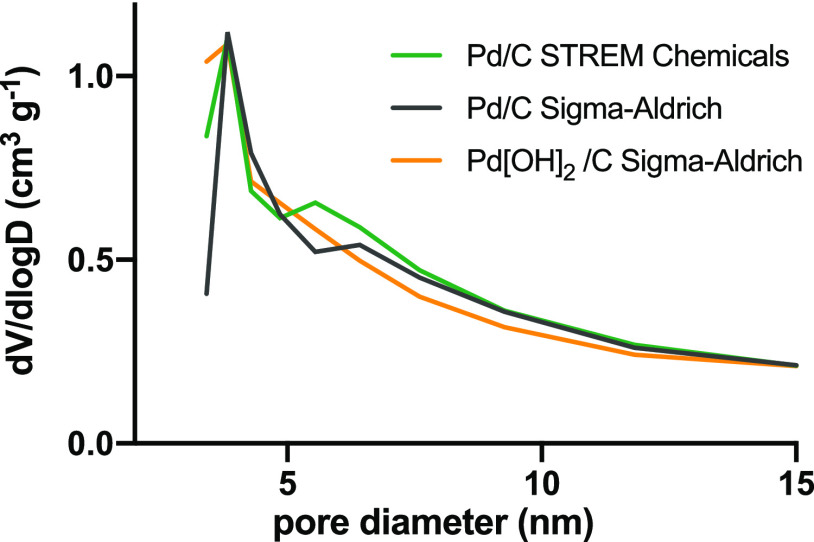
Pore size distribution plots of samples analyzed.

The average pore size distribution (Figure 4) emphasized the presence of mesopores with a mean diameter of about ∼4.0 nm. It can also be seen from the above microstructure data that the specific surface area and pore size distribution of the catalyst are not obviously different, strongly suggesting that the significant difference in catalytic performance reflected by the catalyst mainly comes from the different compositions of its active substance (Pd and PdO).

### Catalyst Recycling Study

**Figure 5:**
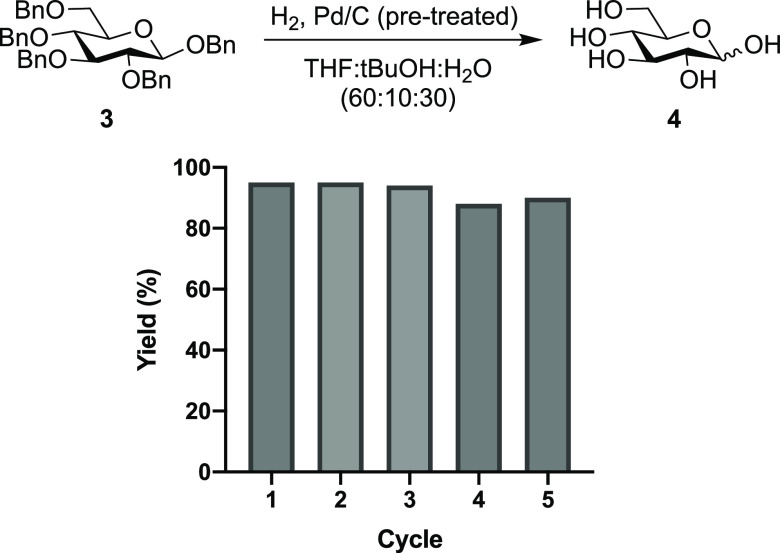
Catalyst recycling of the pretreated catalyst.

The possibility of catalyst recycling was investigated using the pretuned catalyst (3#). Catalyst recycling allows chemists to reduce their use of rare earth metals, reducing waste, and cost.^22^ This may be useful as frequently in oligosaccharide deprotection chemists use high quantities of Pd/C compared to that of “typical hydrogenolysis” reactions, as each Pd catalyst must complete multiple cycles “on one substrate” to give the desired product. We completed our recycling study using per-benzylated glucoside 3 as our model substrate, reisolating the Pd/C catalyst through centrifugation. After five cycles, there was no evidence of catalyst deactivation, loss of activity, and yields of **4** ranged from 95–88% (Figure 5). The pretreated recycled catalyst was then analyzed by XRD and TEM. It was found that the composition of the catalyst had not altered significantly, with XRD confirming the presence of both Pd and PdO (Supporting Information (SI), Figure 1A). TEM imaging (SI, Figure 1B) showed that the small particles of Pd species remained evenly distributed after five cycles, without obvious maturation and agglomeration growth. All supporting that the catalyst experienced no loss of activity after several cycles and suggesting that the pretreatment process does not alter the Pd/C catalyst surface chemistry significantly.

## Conclusions

We found considerable variability in three batches of commercial palladium on carbon catalysts and assayed their physical and chemical properties. In our model system, hydrogenolysis of a decasaccharide, we found a 5% Pd/C from Strem chemicals to be the most effective. Giving the highest yields, shortest reaction times, and minimal levels of saturated impurities. We also demonstrate the possibility of recycling this catalyst after it was subject to our pretuning preparation, which improves the palladium’s selectivity for hydrogenolysis, finding no signs of deactivation or reduced yields. This catalyst was unique in our study, with the finding that this heterogeneous catalyst contained both PdO and Pd^0^. Additionally, its PdO crystals were the smallest observed in all three samples. This ideal catalyst also had smaller Pd^0^ particles and a more uniform distribution on the carbon surface of PdO and Pd^0^.

It is therefore likely that other effective catalysts will contain these physical and chemical properties. Therefore, these analysis techniques are a powerful means to investigate catalyst quality and allows chemists to make a prediction of the quality—prior to the use of valuable synthetic material. Three key findings are that efficient Pd/C catalysts having (1) small Pd/PdO particle size (2) homogeneous distribution of Pd/PdO on the carbon support, and (3) the palladium oxidation state (presence of both Pd^0^ and Pd^2+^) are good predictors of catalytic efficiency.

## Experimental Section

### Synthesis of the Decasaccharide Substrate

The synthesis was reported previously,^11^ using a convergent building block approach, utilizing di- and tetrasaccharide thioglycoside building blocks.^23^

### Procedure for Catalyst Pretreatment^4^

Pd/C (500 mg, any commercial catalyst) was added to a 10 mL round bottom flask, suspended in a DMF/H_2_O mixture (1 mL, 80:20 v/v), and the solution was acidified by the addition of 200 μL of HCl (ACS Reagent, 37%, pH 2–3), with or without an atmosphere of hydrogen gas for about 20 min with vigorous stirring (400 rpm). The presence of dimethylamine was confirmed by ninhydrin staining. The treated Pd/C catalyst was reisolated by filtration. The moistened catalyst was then used directly in the hydrogenolysis reaction.

### Optimized Procedure for the Hydrogenolysis Reaction^4^

The treated catalyst (0.2–0.5 molar equiv of palladium per benzyl group) was added to a solution of oligosaccharide (1 equiv) dissolved in THF:*tert*-butyl alcohol:phosphate-buffered saline (PBS) solution (4 mL, 100 mM, pH 4) (60:10:30, v/v/v). The reaction was placed in a high-pressure reactor at 10 bar with vigorous stirring (400 rpm). The reaction progress was monitored via normal phase thin-layer chromatography (TLC) (MeCN:H_2_O mixtures) and MALDI-TOF mass spectrometry. Once complete, the reaction mixture was filtered through a plug of celite and then concentrated in vacuo. The residue was then redissolved in a minimal amount of sterile water and purified with a Bio-Gel P-2 Column, after lyophilization to yield the desired product.

### Palladium on Carbon Characterization

A transmission electron microscope (JEOL JEM-2100F) was used to obtain the images. High-resolution TEM (HRTEM) images were recorded with an acceleration voltage of 200 kV. An X-ray diffractometer (XRD, Bruker D8 Advance) with Cu Kα radiation was used for analyzing the crystal structure of the as-prepared samples from 5–90° with a scanning step of 0.02°. The surface elemental composition and chemical state of the as-prepared samples were obtained with an X-ray photoelectron spectrometer (XPS, Kratos Axis Ultra DLD), in which a monochromatic Al Kα source (*h*ν = 1486.6 eV) was applied. All binding energies were calibrated using the C 1s hydrocarbon peak at 284.60 eV. The nitrogen sorption measurement was carried out on Quantachrome QUADRASORB evo at liquid nitrogen temperature.

### General Notes

Silica gel flash chromatography was carried out using automated flash chromatography systems, Buchi Reveleris X2 (UV 200–500 nm and ELSD detection, Reveleris silica cartridges 40 μm, BÜCHI Labortechnik AG). Size-exclusion chromatography was performed on Bio-Gel P-2 (Bio-Rad Laboratories Inc.) using isocratic elution (H_2_O/^*t*^BuOH, 99:1, v/v). Instrumentation: peristaltic pump P-3 (Pharmacia Fine Chemicals), refractive index detector Iota 2 (Precision Instruments), and PrepFC fraction collector (Gilson Inc.). Software: Trilution LC (version 1.4, Gilson Inc.). All chemicals for the synthesis were purchased from commercial suppliers and used without purification. Anhydrous solvents were obtained from a PureSolv-ENTM solvent purification system (Innovative Technology Inc.). All other anhydrous solvents were used as purchased from Sigma-Aldrich in AcroSeal bottles.

## Supporting Information Available

Additional analysis (TEM and XRD) of Pd/C catalysts used in this study (PDF)
