# Supplementary material for: Defining the Qualities of High-Quality Palladium on Carbon Catalysts for Hydrogenolysis
Source: Org Process Res Dev. 2021 Jun 23;25(7):1573–8. doi: 10.1021/acs.oprd.0c00536 (PMC8291771; doi:10.1021/acs.oprd.0c00536)

Supporting Information

Defining the qualities of high-quality palladium on carbon catalysts for hydrogenolysis

Conor J. Crawford,[a,b]* Yan Qiao,[c,d] Yequn Liu,[c,d] Dongmei Huang,[c,d] Wenjun Yan,[c,d], Peter H. Seeberger,[b] Stefan Oscarson,[a]* and Shuai Chen,[d]*

[a] Centre for Synthesis and Chemical Biology, University College Dublin, Belfield, Dublin, Ireland. E-mail: conor.crawford@ucdconnect.ie stefan.oscarson@ucd.ie

[b] Department of Biomolecular Systems, Max Planck Institute of Colloids and Interfaces, 14476 Potsdam, Germany.

[c] Center of Materials Science and Optoelectronics Engineering, University of Chinese Academy of Sciences, Beijing 100049, People's Republic of China.

[d] State Key Laboratory of Coal Conversion, Institute of Coal Chemistry, Chinese Academy of Sciences, Taiyuan 030001, People's Republic of China. Email: chenshuai@sxicc.ac.cn

Table of Contents

TEM and XRD of 5% Pd/C (pre-treated) recycled catalyst 3

Figure S1. TEM and XRD of 5% Pd/C (pre-treated) recycled catalyst 3

TEM of 5% Pd/C STREM Chemicals (1#) 4

TEM 10% Pd/C Sigma-Aldrich (2#) 9

TEM of 20% $Pd[OH]_2/C$ Sigma-Aldrich (3#). 15

Palladium: PDF 05-0681 20

Palladium oxide: PDF 88-2434 21

SEM-EDS 22

TEM and XRD of 5% Pd/C (pre-treated) recycled catalyst

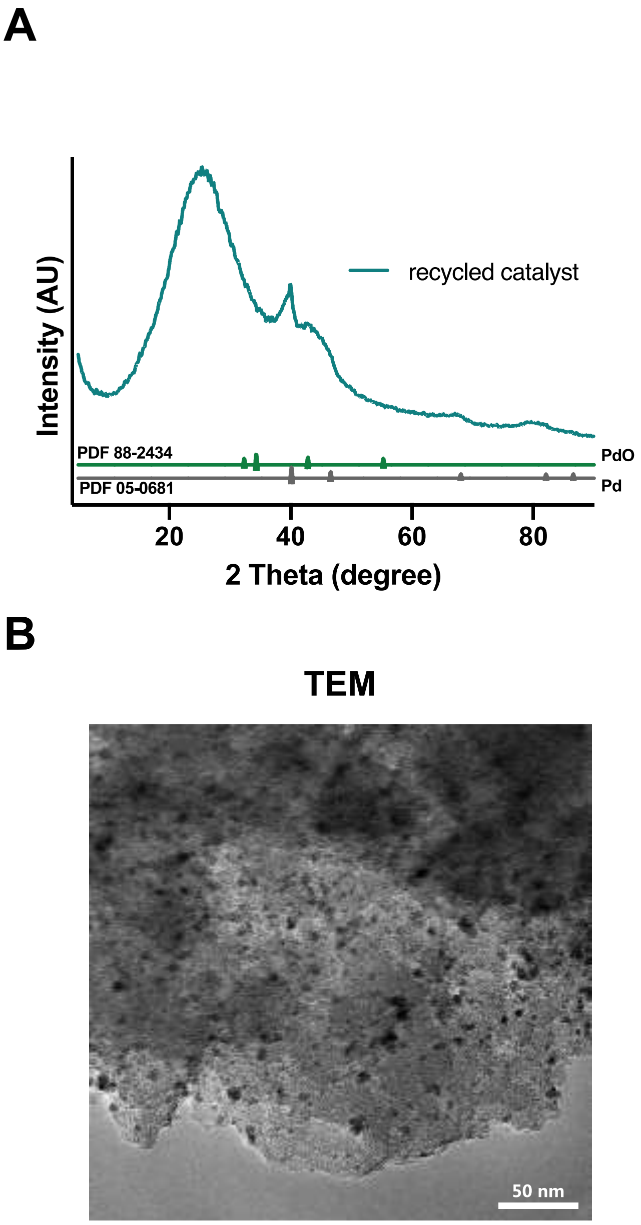

Figure S1. TEM and XRD of 5% Pd/C (pre-treated) recycled catalyst

TEM of 5% Pd/C STREM Chemicals (1#)

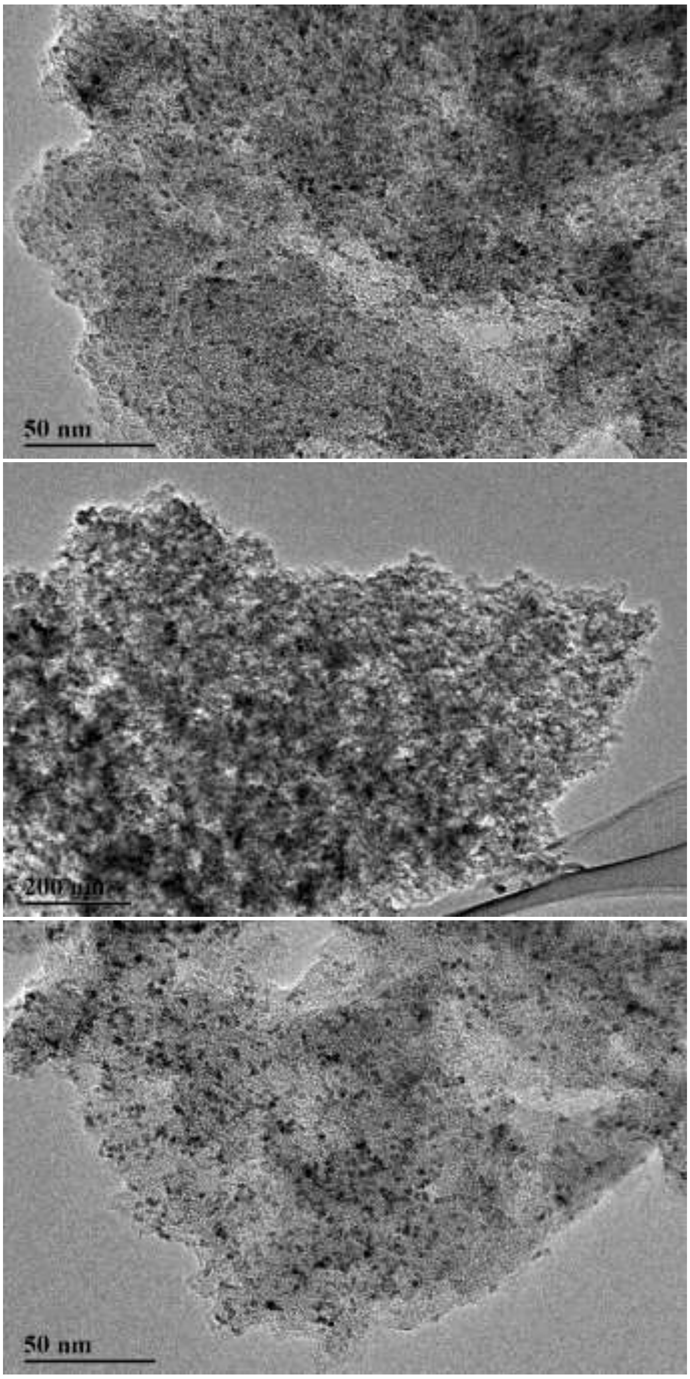

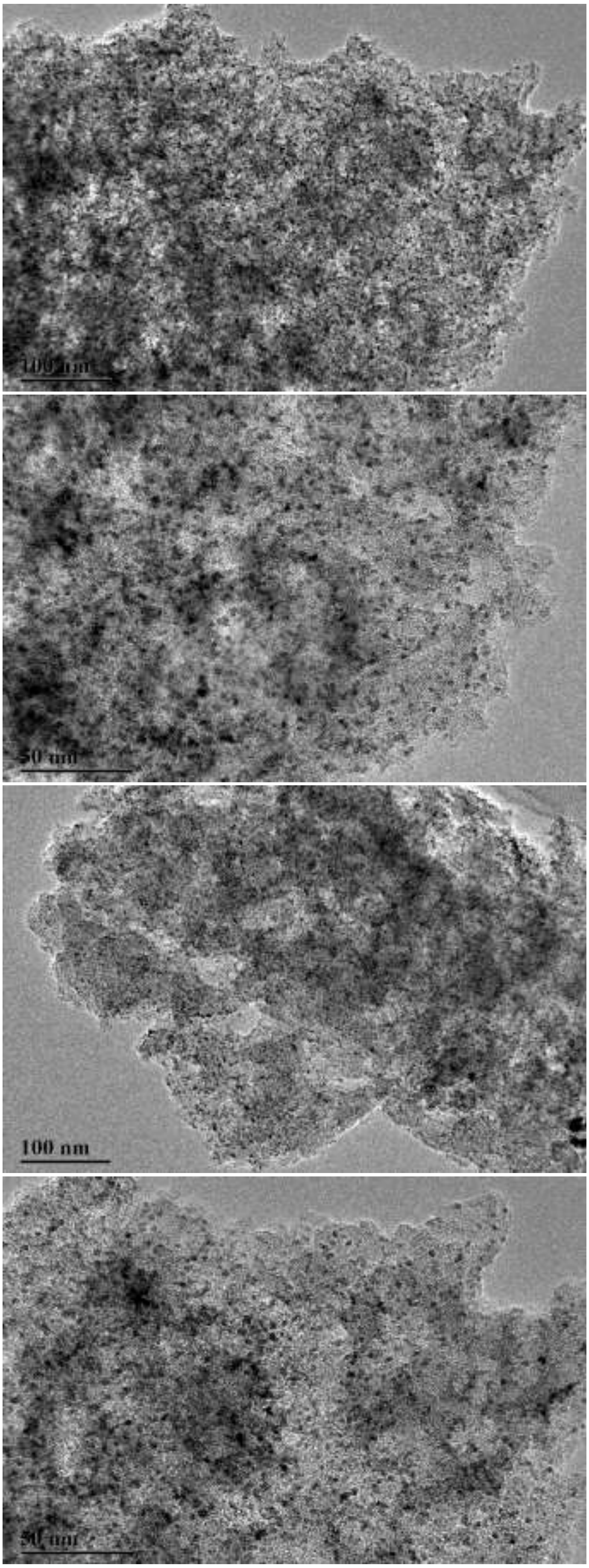
100 nm
50 nm
100 nm
50 nm

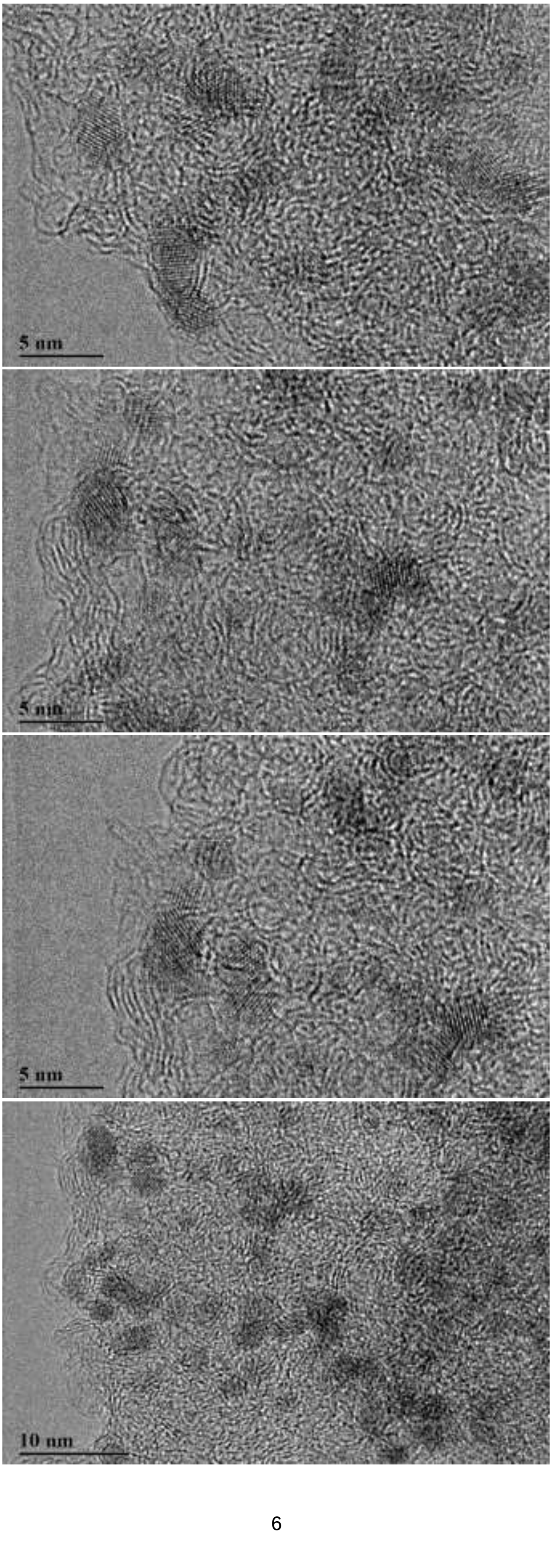
5 nm
5 nm
5 nm
10 nm

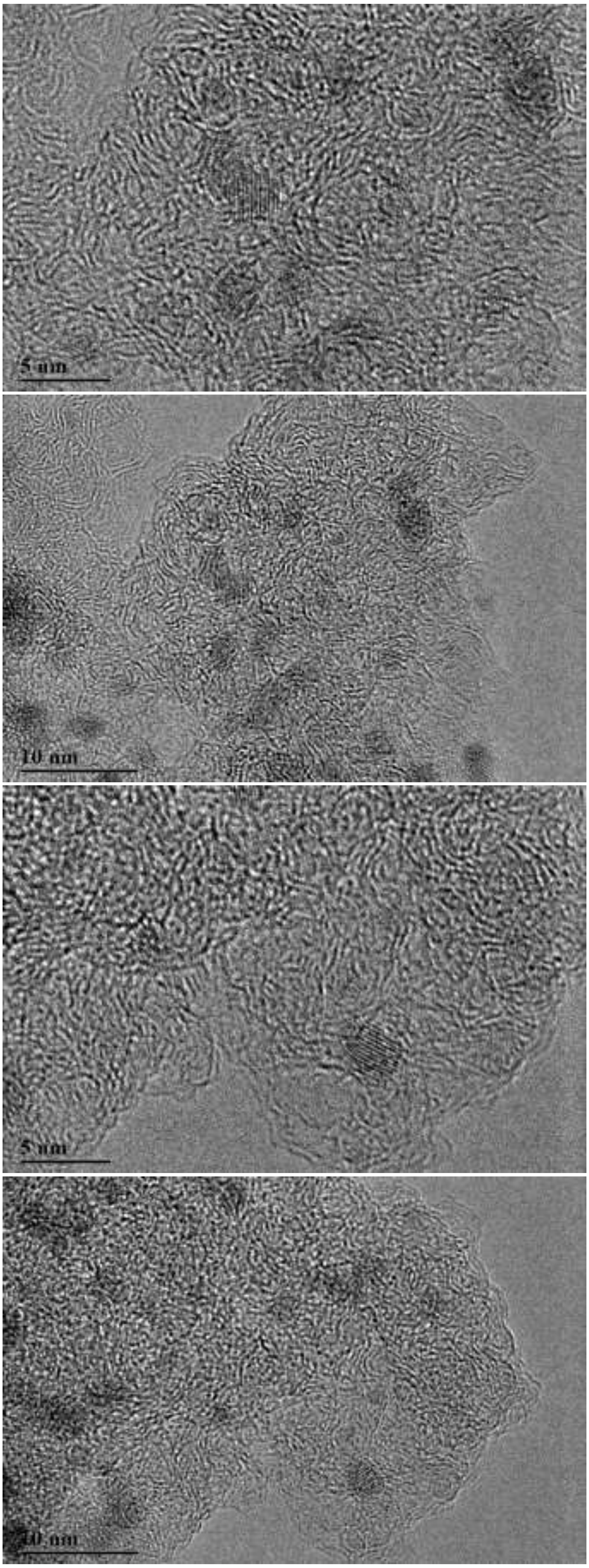
5 nm
10 nm
5 nm
10 nm

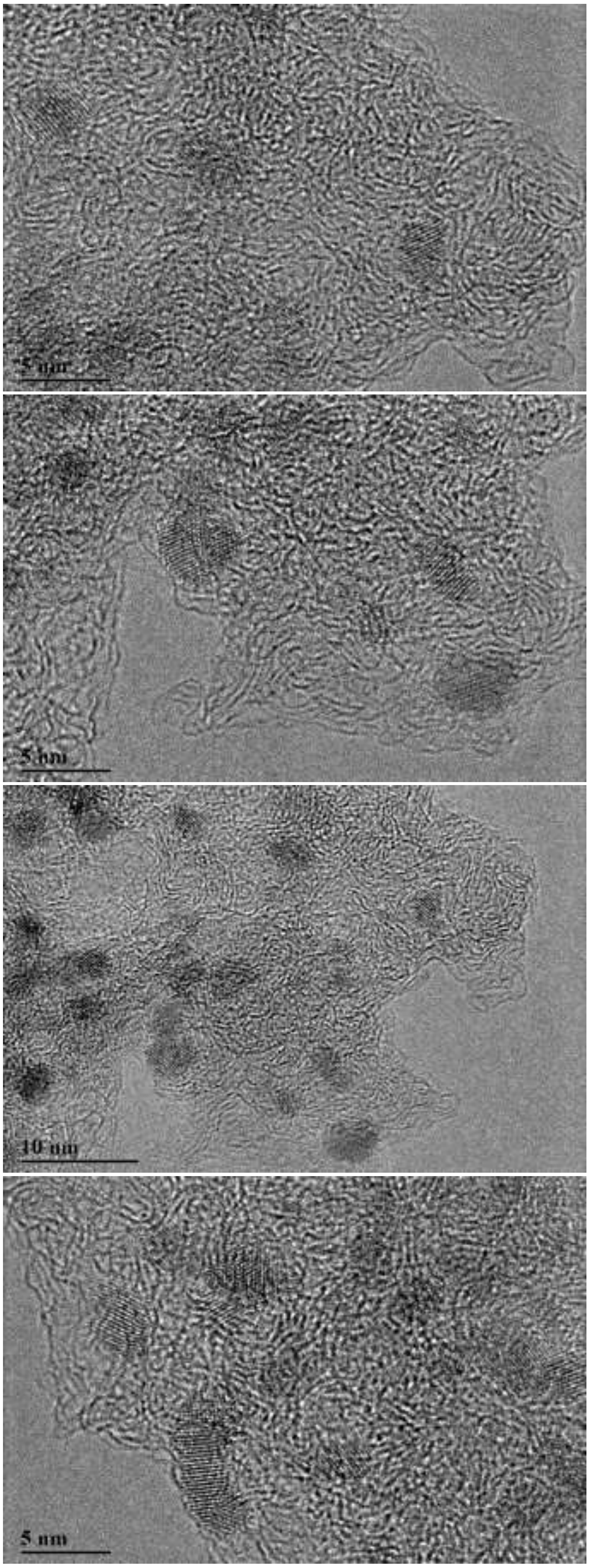
5 nm
5 nm
10 nm
5 nm

TEM 10% Pd/C Sigma-Aldrich (2#)

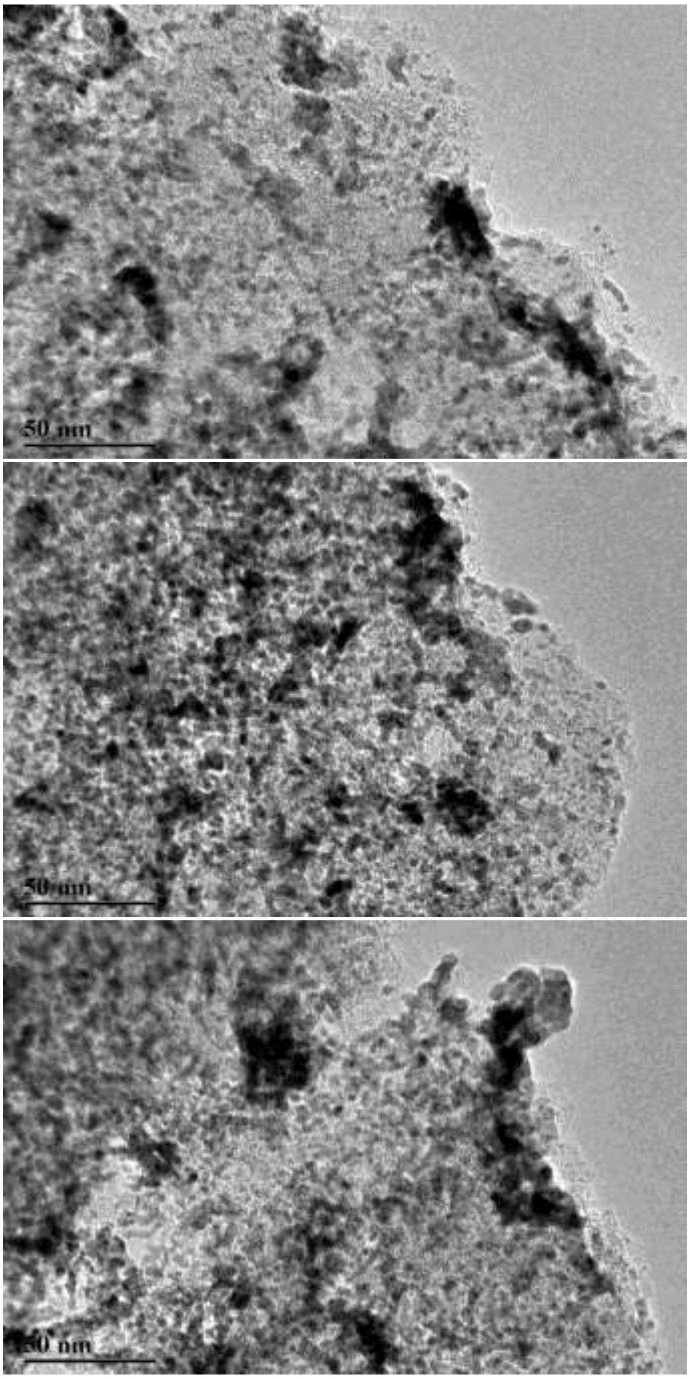

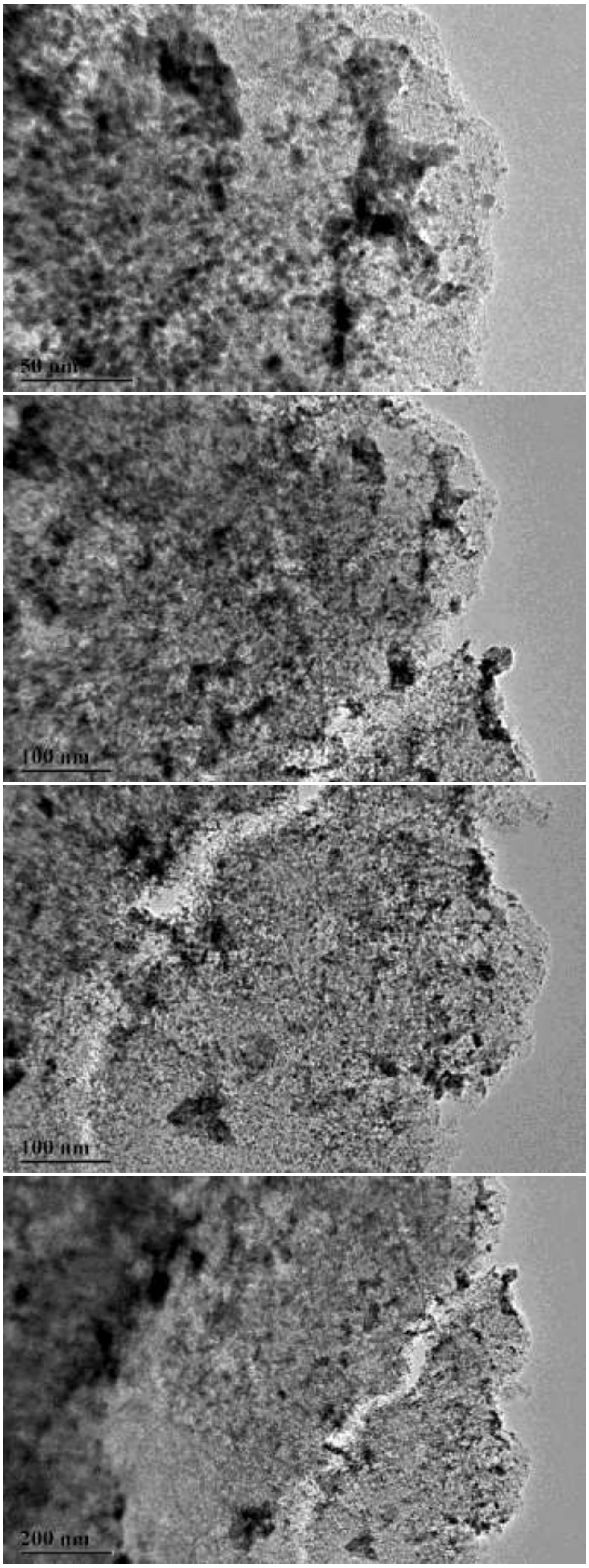
50 nm
100 nm
100 nm
200 nm

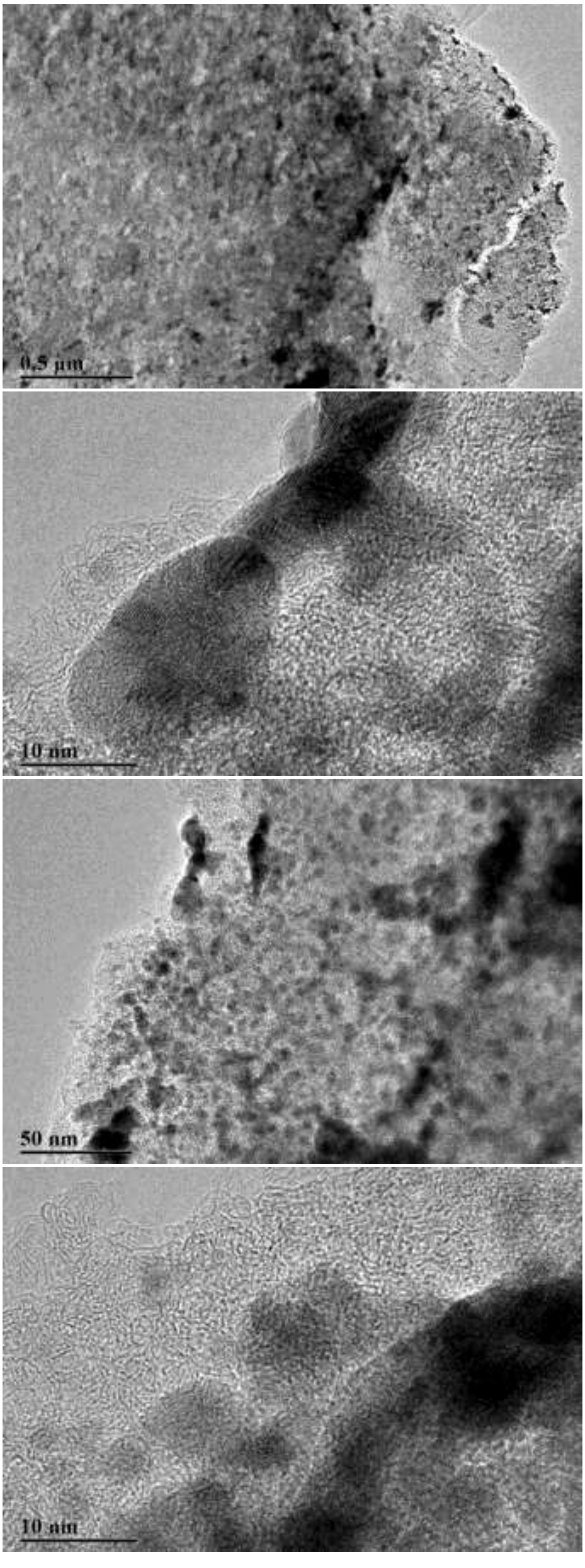
0,5 µm
10 nm
50 nm
10 nm

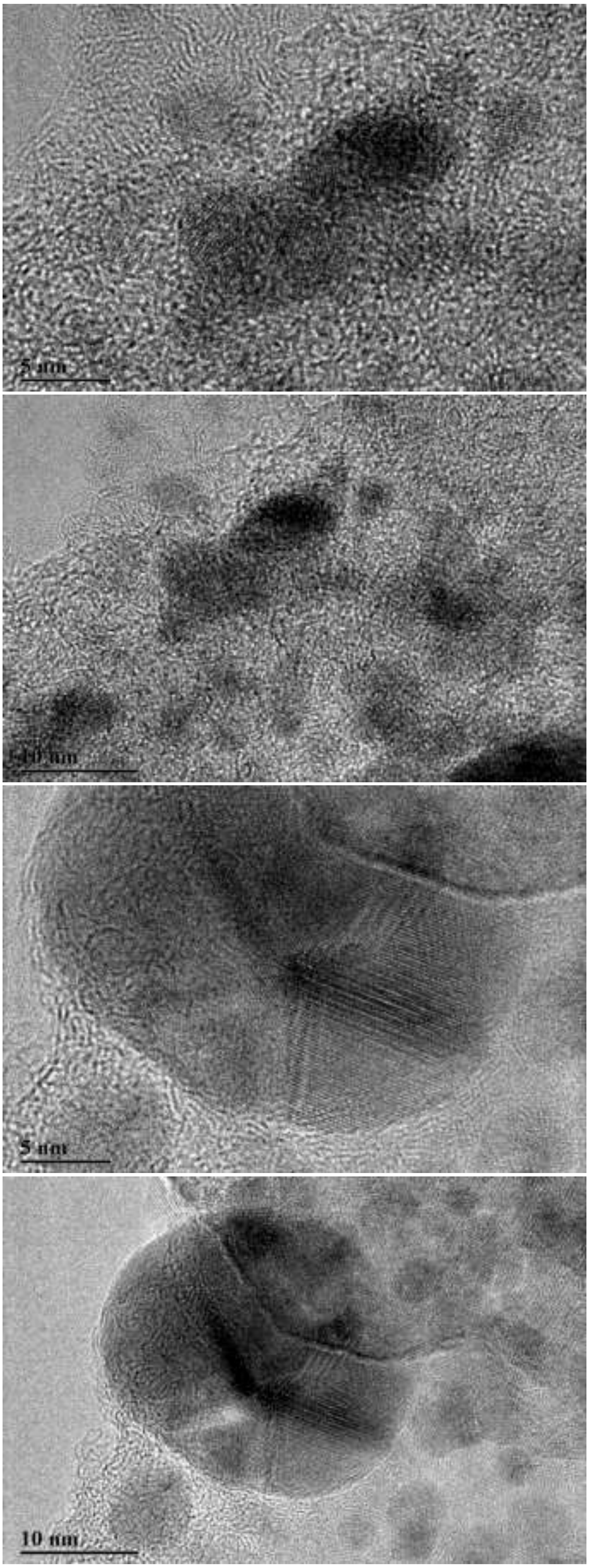
5 nm
10 nm
5 nm
10 nm

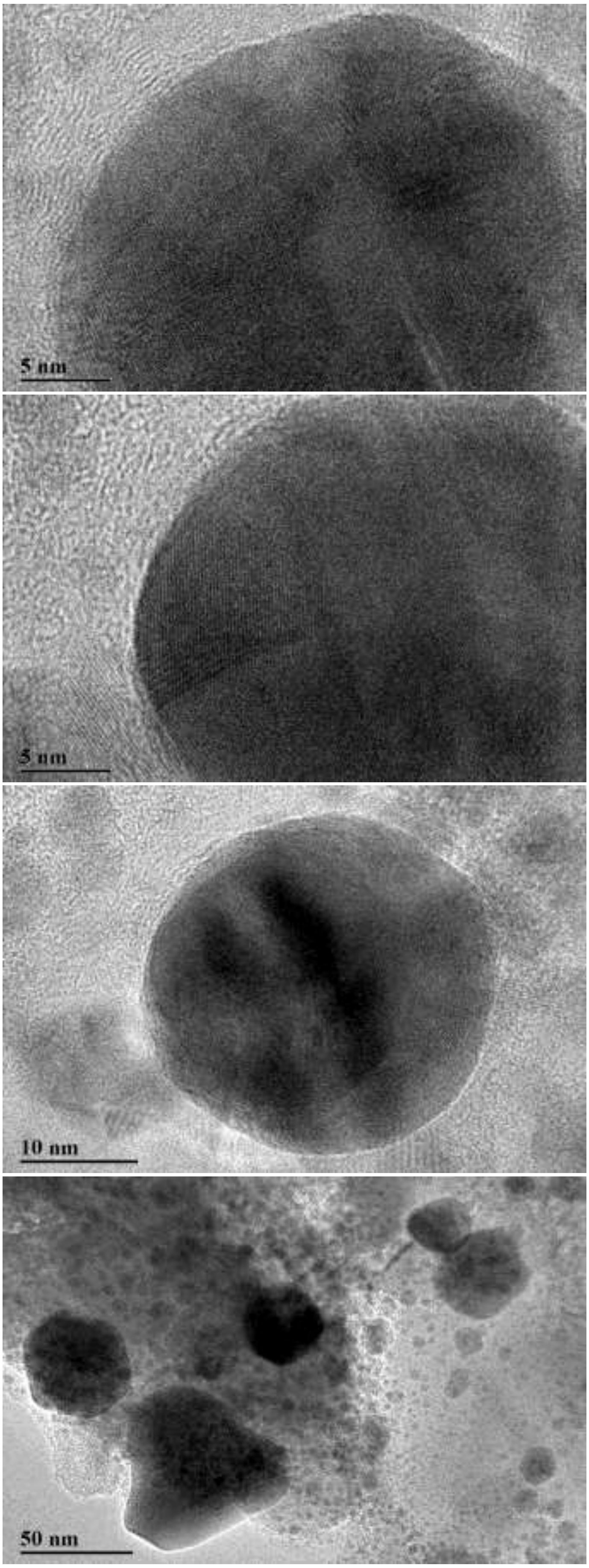
5 nm
5 nm
10 nm
50 nm

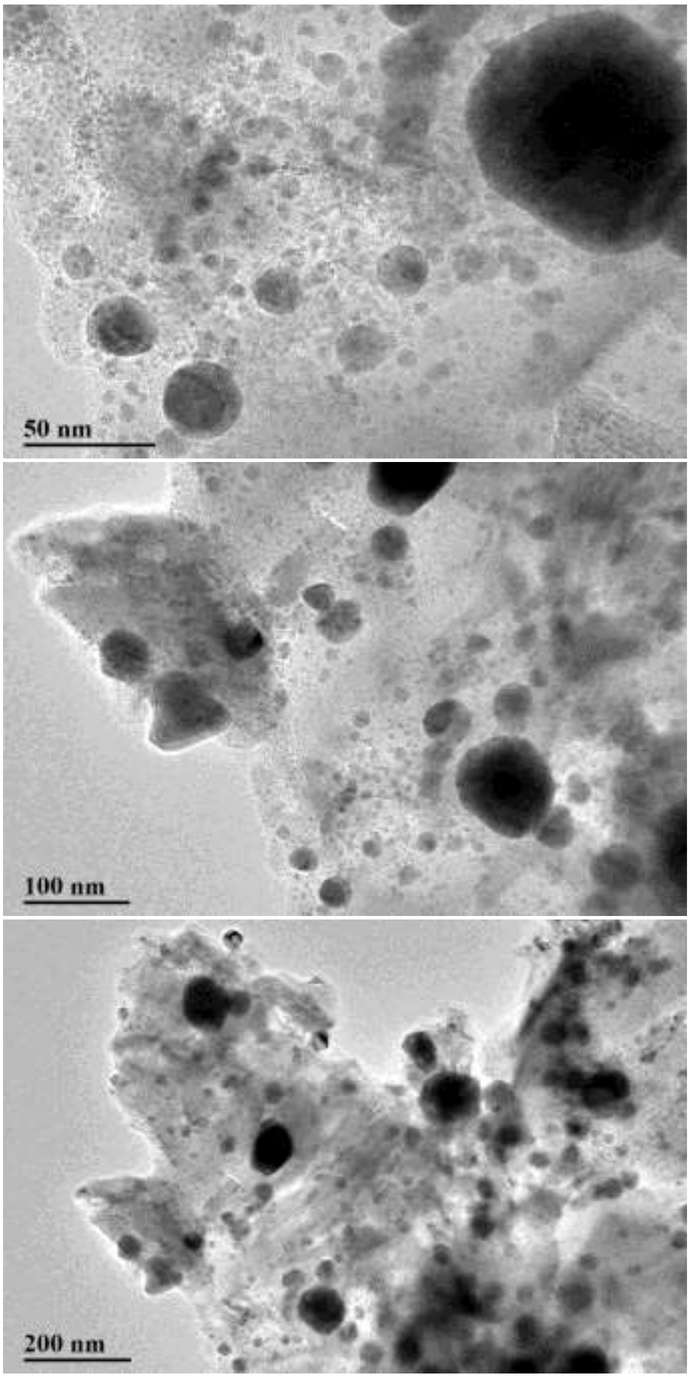
50 nm
100 nm
200 nm

TEM of 20% $Pd[OH]_2/C$ Sigma-Aldrich (3#).

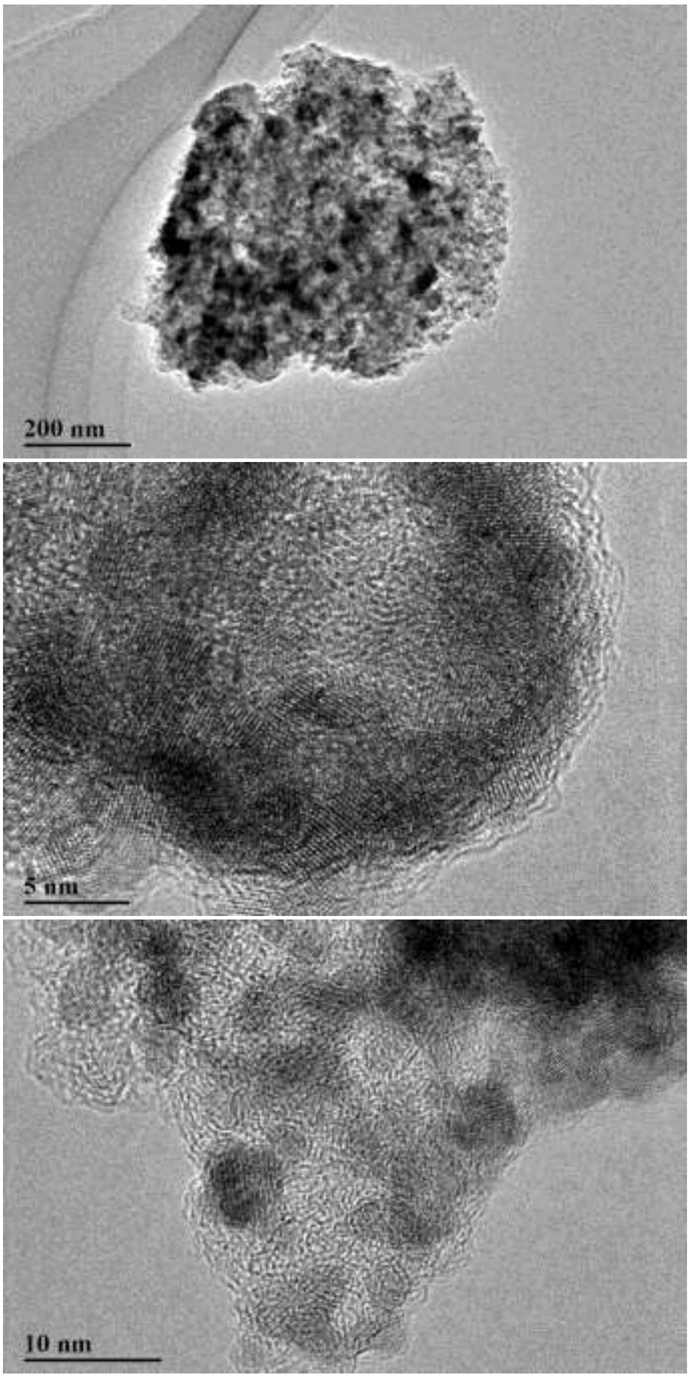

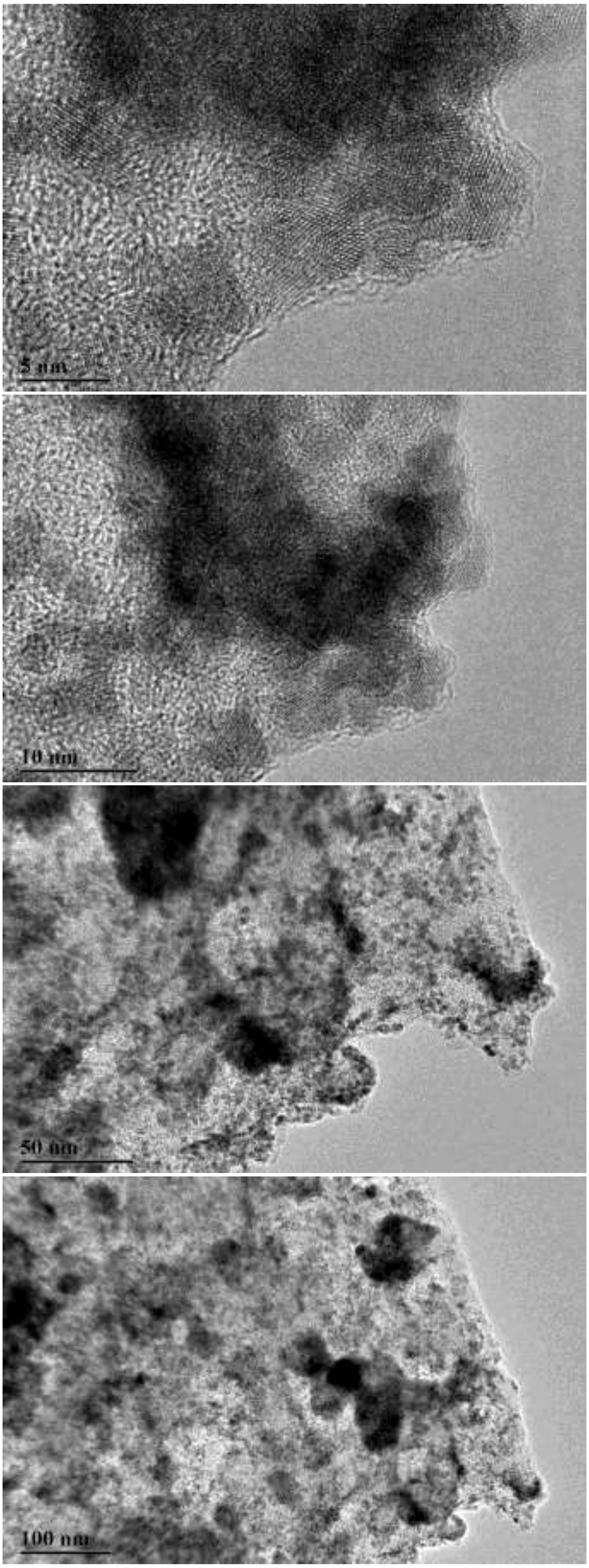
5 nm
10 nm
50 nm
100 nm

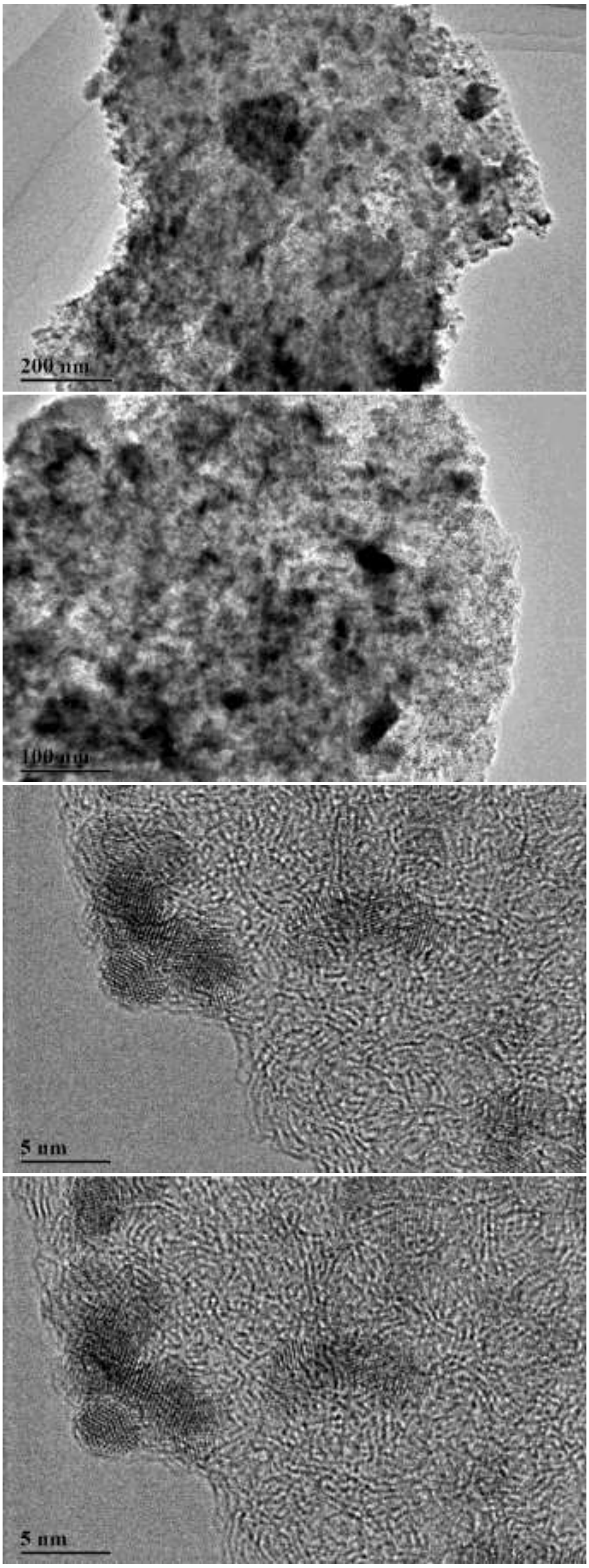
200 nm
100 nm
5 nm
5 nm

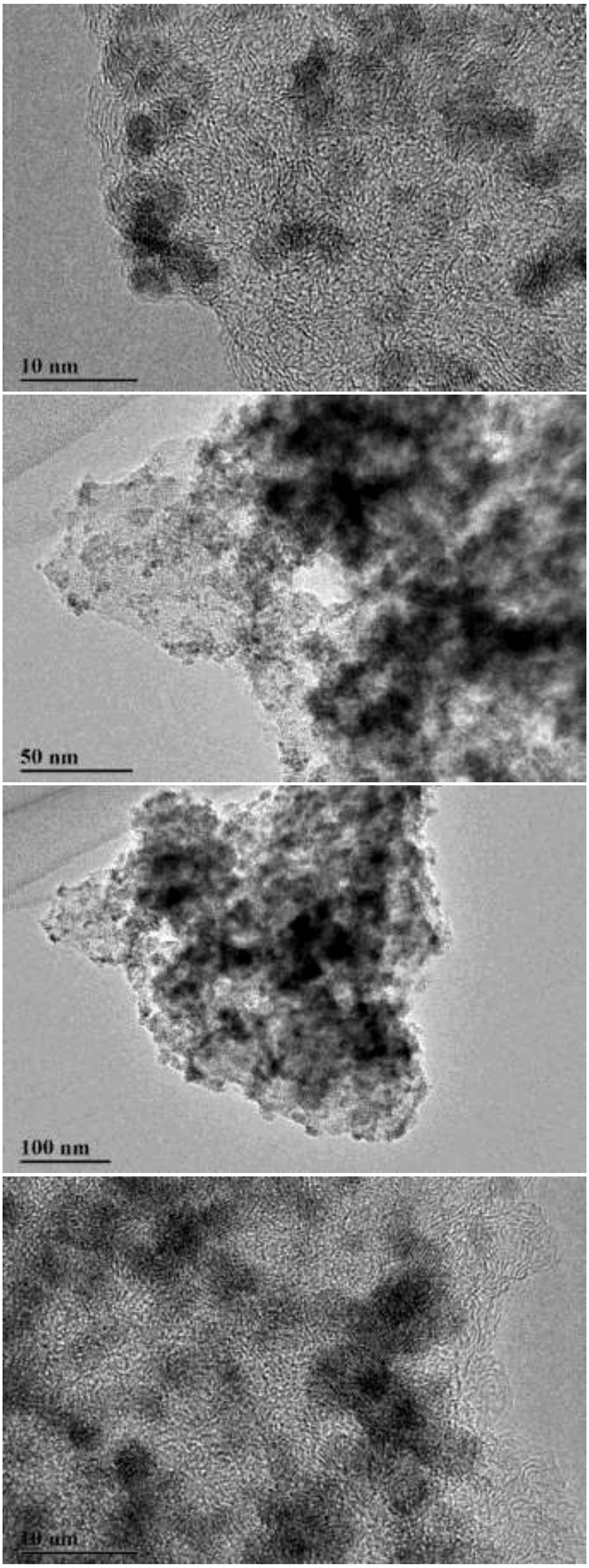
10 nm
50 nm
100 nm

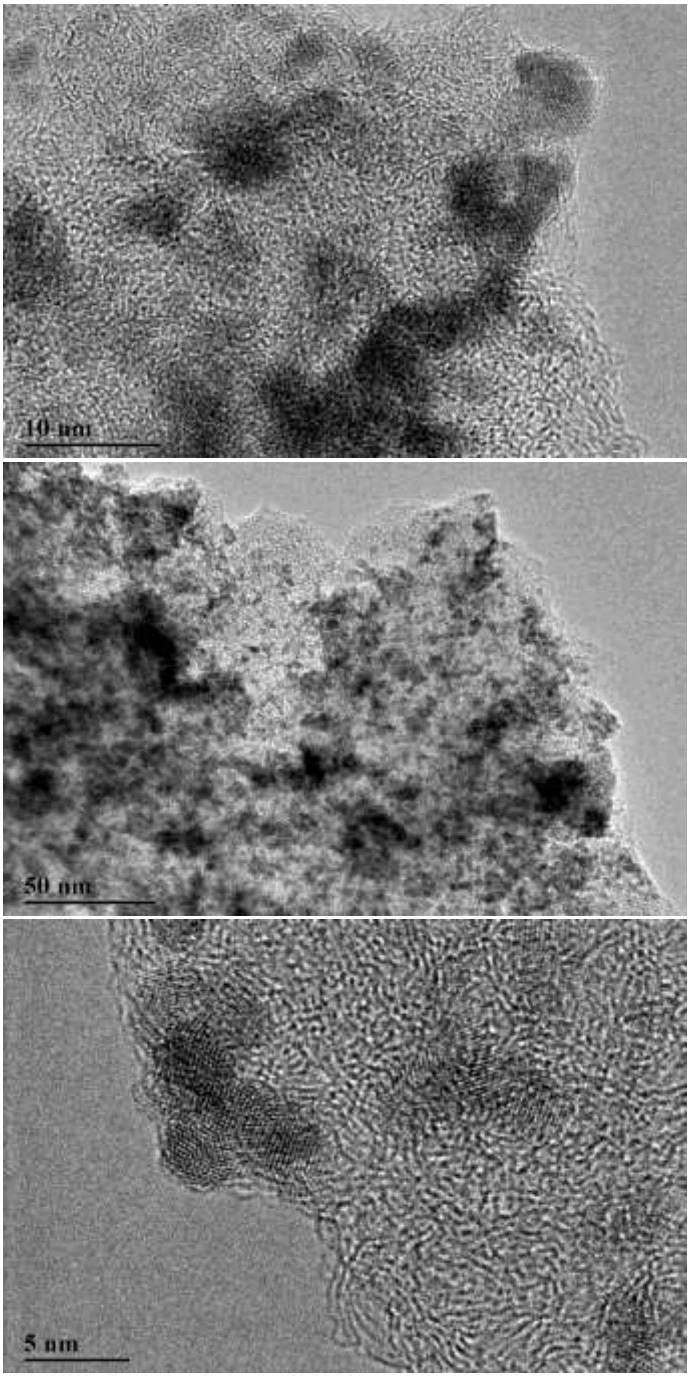
10 nm
50 nm
5 nm

Palladium: PDF 05-0681

Pattern: PDF 05-0681 Radiation: 1.54060 Quality: Star (*)

Formula	Pd
Name	Palladium
Name (mineral)	Palladium, syn
Name (common)	

Lattice:	Cubic	Mol. weight =	106.4
S.G.:	Fm-3m (225)	Volume [CD] =	58.85
		Dx =	
		Dm =	
		I/Icor =	-1.000

a =	3.88980	alpha =	
b =		beta =	
c =		gamma =	
a/b =	1.00000	Z =	4
c/b =	1.00000		

Color: Black
Sample Source Or Locality: Sample from Johnson Matthey Company, Ltd
Analysis: Spectroscopic analysis shows <0.1% Ag, Si; <0.01% Ca, Cu, Mg, Pt; 0.0001% Pb
Temperature Of Data Collection: Pattern taken at 26 C
Deleted By or Rejected By: Deleted by 46-1043; F#N is higher, Mayo 11/94

Primary Reference
Publication: Natl. Bur. Stand. (U.S.), Circ. 539
Detail: volume I, page 21 (1953)
Authors: Swanson, Tatge.

Radiation:	CuKa1	Filter:	F
Wavelength:	1.54060	d-spacing:	
SS/FOM:	55.9 (0.0179,8)		

d	2θ	I fix	h	k	l
2.24600	40.115	101	1	1	1
1.94500	46.662	43	2	0	0
1.37600	68.085	26	2	2	0
1.17300	82.096	25	3	1	1
1.12320	86.598	9	2	2	2
0.97230	104.792	4	4	0	0
0.89240	119.351	14	3	3	1
0.86970	124.677	12	4	2	0

Palladium oxide: PDF 88-2434

Pattern: PDF 88-2434 Radiation: 1.54060 Quality: Calculated

Formula Pd O
Name Palladium Oxide
Name (mineral)
Name (common) Palladium oxide - HP

Lattice: Tetragonal
S.G.: I4/mmm (139)

Mol. weight = 122.4
Volume [CD] = 47.87
Dx =
Dm =
I/Icor = 10.080

a = 2.98200
b =
c = 5.38300
a/b = 1.00000
c/b = 1.80516

alpha =
beta =
gamma =
Z = 2

ICSD Collection Code: 041617
Remark From ICSD/CSD: REM M Stable above 12 GPa, elongated rocksalt-type
Remark From ICSD/CSD: REM M Cell at ambient pressure 3.042, 5.351, cp. 24692
Remark From ICSD/CSD: REM M PDF 43-1024
Test From ICSD: No R value given
Test From ICSD: At least one TF missing
Remark From ICSD/CSD: REM PRE Mentioned
Article Title: Structural behavior of palladium(II) oxide and a palladium suboxide at high pressure: an energy-dispersive X-raydiffraction study
Wyckoff Sequence: b a (I4/MMM)
ANX: AX

Structure
Publication: Phys. Rev. B: Condens. Matter
Detail: volume 52, page 9259 (1995)
Authors: Christy, A.G., Clark, S.M.
Primary Reference
Publication: Calculated from ICSD using POWD-12++

Radiation: CuKa1
Wavelength: 1.54060
SS/FOM: 999.9 (0,10)

Filter: Not specified
d-spacing:

d	2θ	I fix	h	k	l
2.69150	33.261	515	0	0	2
2.60849	34.352	999	1	0	1
2.10859	42.854	438	1	1	0
1.65987	55.300	342	1	1	2
1.53746	60.135	148	1	0	3
1.49100	62.213	108	2	0	0
1.34575	69.835	34	0	0	4
1.30425	72.400	117	2	0	2
1.29446	73.036	144	2	1	1
1.13440	85.537	62	1	1	4

SEM-EDS

1# Sample, Pd/C STREM Chemicals: Cl=1.7%$_{wt}$

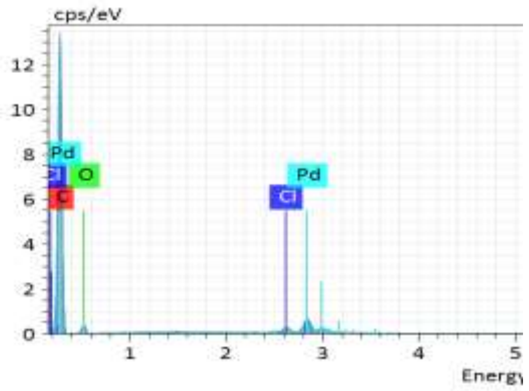

2# Sample, Pd/C Sigma-Aldrich: Cl=0.8%$_{wt}$

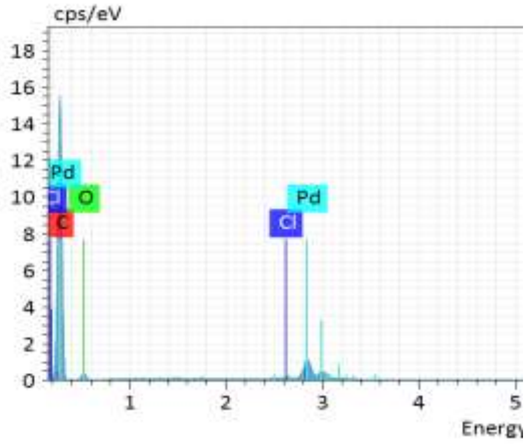

3# Sample, Pd[OH]$_2$ /C Sigma-Aldrich: negligible

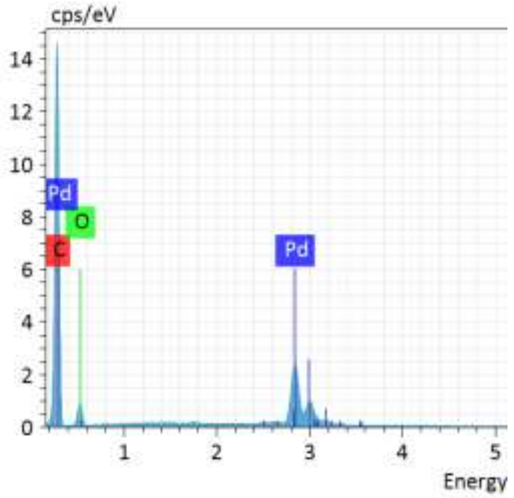

Supplement: Supplementary file 1 — op0c00536_si_001.pdf [file op0c00536_si_001.pdf]
